# The Efficacy of Puromycin and Adriamycin for Induction of Glomerular Failure in Larval Zebrafish Validated by an Assay of Glomerular Permeability Dynamics

**DOI:** 10.1089/zeb.2017.1527

**Published:** 2018-06-01

**Authors:** Sebastien Andrew Rider, Finnius Austin Bruton, Richard George Collins, Bryan Ronald Conway, John James Mullins

**Affiliations:** ^1^ Univeristy/BHF Centre for Cardiovascular Science, The Queen's Medical Research Institute, Little France, The University of Edinburgh, Edinburgh, United Kingdom.; ^2^ Edinburgh College of Art, University of Edinburgh, Edinburgh, United Kingdom.

**Keywords:** puromycin, adriamycin, podocyte, glomerulus, pharmacological, kidney

## Abstract

Defects in the glomerular filtration barrier (GFB) play a major role in the onset of human renal diseases. Highly ramified glomerular cells named podocytes are a critical component of the GFB. Injury to podocytes results in abnormal excretion of plasma proteins, which can lead to chronic kidney disease. The conserved paired nephron of larval zebrafish is an excellent model for assessing glomerular function and injury. The efficacy of two known podocyte toxins was tested to refine models of acute podocyte injury in larval zebrafish. The validated compound was then used to test a novel assay of the dynamics of abnormal protein excretion. Injected adriamycin was found to be unsuitable for induction of glomerular injury due to off-target cardiovascular toxicity. In contrast, puromycin treatment resulted in a loss of discriminative filtration, measured by excretion of 70 kDa dextran, and podocyte effacement confirmed by electron microscopy. The dynamics of dextran excretion during puromycin injury modeled the onset of glomerular damage within 24 hours postinjection. These data validate puromycin for induction of acute podocyte injury in zebrafish larvae and describe a semihigh-throughput assay for quantifying the dynamics of abnormal protein excretion.

## Introduction

Approximately 1 million glomeruli filter 90 L of blood in each human kidney every day.^1^ The precise filtration of blood constituents depends on the maintained integrity of the glomerular filtration barrier (GFB). The GFB comprises two highly specialized cell types separated by a type IV collagen and laminin-rich glomerular basement membrane (GBM).^2^ The fenestrated endothelium forming the walls of glomerular capillaries is enwrapped by podocytes. Podocytes are highly ramified epithelial cells with primary and secondary foot processes, the structure of which depends on their actin cytoskeleton.^3^ The filtration slits between interdigitated podocyte foot processes comprise highly specialized gap junctions called slit diaphragms (SDs). The SD protein complex forms the barrier to protein filtration and is essential for maintained podocyte structure and function.^4^

All identified monogenic mutations associated with nephrotic syndrome are in podocyte-specific genes, revealing the essential role of these specialized cells for glomerular function. Disease-causing recessive or dominant autosomal mutations have been identified, among others, in *nephrin* (*NPHS1*),^5^ *transient receptor potential channel 6* (*TRPC6*),^6^ *Wilm's tumor suppressor gene 1* (*WT1*),^7^ and *podocin* (*NPHS2*).^8^ Most glomerular injuries are, however, not hereditary, but acquired in immune or nonimmune diseases, with 75% of cases having an unknown cause.^9^

Podocyte effacement, the gradual simplification of the interdigitating foot process pattern, is a key pathology common to most glomerular diseases.^9,10^ The breakdown of the GFB in disease, or as a result of drug toxicity, causes proteinuria, the abnormal filtration of plasma proteins, including albumin. Proteinuria associated with nephrotic syndrome results in edema, likely due to increased sodium retention by the distal nephron.^11^

Experimental animal models are required that recapitulate the molecular events that manifest during glomerular injury and the sequelae of pathological responses that lead to glomerulosclerosis and the loss of functioning glomeruli.^9^ In rodents, acute nephrosis may be experimentally induced through insult to podocytes by nephrotoxic compounds. Injection of the antibiotic, puromycin aminonucleoside (PAN), causes either a mild or reversible glomerular damage resembling minimal change disease, or more severe lesions that mimic human focal segmental glomerulosclerosis (FSGS),^12,13^ the latter a hallmark of lasting and progressively damaging chronic kidney diseases.^14^ Similarly, a single injection of the chemotherapy drug, adriamycin, causes a severe form of FSGS in rats and mice.^15^

With its unique set of experimental advantages, including genetic tractability and unparalleled opportunity for *in vivo* experimentation, zebrafish have become a valuable animal model for renal research. The small size, rapid development, and translucency of larval zebrafish offer excellent opportunities for whole-animal chemical and genetic screening^16^ and, recently, for studies of renal cancer.^17–21^ Within the first 3 days of life, the pronephros of larval fish becomes functional and capable of discriminative filtration.^22,23^ Mature pronephric podocytes in larval zebrafish are anatomically similar to their mammalian counterparts and are nonmigratory, including during repair.^24–26^ With a developed glomerulus, segmented tubules, and renin-expressing cells, the single paired nephron of the zebrafish pronephros shares remarkable anatomical and functional similarity with the metanephric mammalian nephron.^22,23,27–33^

Due to inherent limitations in current methods, the zebrafish model requires novel and refined renal injury techniqiues.^34^ A particular drawback to pharmacological models of acute renal failure in larval fish is that they are terminal, which limits the potential for follow-up studies.^34^ Genetic approaches for induction of glomerular injury have successfully utilized nitroreductase (NTR)^35–37^ to induce ablation of podocytes by cytotoxic metabolization of metronidazole (MTZ).^38^ This recapitulates proteinuria and has been successfully used to demonstrate the regenerative capacity of fish podocytes postinjury.^25,35,37^ However, the NTR/MTZ system is limited by side effects of MTZ and the prolonged time required for induction of apoptosis.

While also often limited by off-target effects, pharmacological approaches are quick to implement and may be applied to a variety of wild-type or transgenic lines. Current methods for the pharmacological disruption of podocytes in zebrafish utilizing puromycin result in an increased glomerular permeability.^39^ A comparison of pharmacological agents for induction of podocyte injury in zebrafish is required to identify compounds exhibiting the greatest potency with the lowest off-target effects.

The aims of the present study were twofold, (1) to compare puromycin and adriamycin for pharmacological induction of glomerular injury in zebrafish larvae with an established GFB and (2) to develop a high-throughput assay to measure the *in vivo* dynamics of abnormal protein excretion.

Adriamycin and puromycin were tested for their ability to induce structural podocyte damage and increased glomerular permeability. Increased permeability was determined by the excretion of normally retained dextran, and changes in podocyte morphology were assessed by transmission electron microscopy (TEM). As glomerular function is tightly associated with cardiovascular function in larval fish,^40^ the effects of these compounds on heart rate and blood flow were also tested. After validating a puromycin model of glomerular injury, a novel dextran excretion assay was developed to assay the dynamics of glomerular permeability. Our study provides both a defined pharmacological model of acute podocyte injury and a novel assay enabling the measurement of protein excretion dynamics during glomerular injury and repair.

## Materials and Methods

### Zebrafish and husbandry

All experiments utilizing fish >5 dpf (days postfertilization) were approved by the local ethics committee and conducted in accordance with the Animals (Scientific Procedures) Act 1986 in a UK Home Office-approved establishment. Unless stated otherwise, wild-type WIK zebrafish (*Danio rerio*) were maintained at 28.5°C in 1 × conditioned water (CW)^40^ containing 0.1% (w/v) methylene blue. Where required, fish were anesthetized in CW containing 40 μg mL^−1^ MS-222 (tricaine methanesulfonate). Forty micrograms per milliliter MS-222 is sufficient for anesthesia without significant effects on the heart rate.^41^ Microinjections and microscopy were carried out at room temperature (26°C ± 0.5°C).

### Microinjection of dextran and pharmacological agents

Dextran and pharmacological agents were administered under anesthesia by intravenous microinjection through the cardiac sinus venosus (SV) that drains the common cardinal vein (CCV).^42^ All injections were administered in a 1-nL volume. A successful injection was determined as a 100% delivery into the cardiovascular system. Injections where solutions ended up in the yolk, or in the pericardial sac, were deemed as unsuccessful; 5% (w/v) 70 kDa FITC-dextran was dissolved in phosphate-buffered saline (PBS). PAN (#15509; Cayman Chemical Company) was administered at 5 or 25 mg mL^−1^ (17.0–85.0 mM) and adriamycin (doxorubicin hydrochloride) (#15007; Cayman Chemical Company) at 1, 2, or 4 mg mL^−1^ (1.7, 3.4, 6.9 mM). Shams were administered with vehicle (PBS) only.

### Determination of the dose for induction of glomerular injury

Three dpf fish were injected respective treatment compounds, or vehicle (PBS), and separated into Petri dishes in triplicate. Fish were examined immediately after injection and at 24 and 48 hpi (hours postinjection). At each time point, fish were scored for percentage survival and overt pericardial sac edema.

### Dextran clearance assay

The clearance of 70 kDa FITC-dextran was determined by measuring fluorescence intensity in the dorsal aorta.

In summary, postinjection, fish recovered in CW (without methylene blue) before brief anesthesia and mounting in 3% methylcellulose for baseline imaging at 3 hpi. Sagittal images of FITC fluorescence in the dorsal aorta were taken using a stereomicroscope with fixed parameters. Embryos were returned to CW and standard conditions immediately after each imaging time point. For each fish, the mean intensity of three areas of the dorsal aorta was measured using Fiji—National Institutes of Health (NIH). Mean fluorescence intensity values were normalized against the baseline fluorescence and percentage intensity change was calculated.

### Heart rate and venous erythrocyte velocity

After respective treatments, 3 dpf embryos were left to recover in CW for three hours before mounting in 3% methylcellulose and immediate imaging under anesthesia. Bright-field videos over 120 s in length were acquired of the dorsal aorta at 50 frames per second. Single erythrocytes were then tracked through the cardinal vein anterior of the cloaca. The distance traveled by three erythrocytes per embryo was measured using Fiji and venous erythrocyte velocity calculated in μm s^−1^. Heart rate was assessed similarly by recording the video at a frame rate of 10 frames per second and heartbeats counted manually over a 10-s period. Both videos were taken of a lateral orientation.

### Dextran excretion assay in a modified 96-well plate

Postinjection, fish were left to recover for 1 h in CW (without methylene blue) before distribution into a modified black, flat-bottomed, 96-well Microfluor 1^®^ plate (Nunc). Two hundred fifty-micrometer slits were used to create 48 paired wells (Fig. 4A). In each well pair, one well contained a single fish, leaving the second well free for measurements of FITC intensity in the CW (Fig. 4B). The two well pairs were filled with ∼790 μL CW (without methylene blue) and covered with real-time polymerase chain reaction (qPCR) (Sarstedt) film, leaving no air gap between the water and film. FITC intensity was read once every 15 min for 48 h at a λ_ex_ of 485 nm and λ_em_ of 535 nm. At the end of each assay, fish survival was confirmed.

### TEM and image analysis

Three dpf fish were injected with 25 mg mL^−1^ puromycin or vehicle. At 8 and 24 hpi, fish were fixed for TEM as described by Lyons *et al.*^43^ Briefly, fish were fixed in modified Karnovsky's solution (2% glutaraldehyde and 4% paraformaldehyde in 0.1 M sodium cacodylate buffer), postfixed in 2% osmium tetroxide in 0.1 M imidazole and 0.1 M sodium cacodylate, stained en bloc in saturated uranyl acetate, and dehydrated into 100% acetone. All of these steps were accelerated using microwave stimulation with a Panasonic microwave with inverter technology. Temperature throughout was maintained at 15°C in a cooled water bath. Following dehydration, embryos were embedded in Epon. Ultrathin sections (60 nm thick) were cut from selected areas and mounted on Formvar/carbon-coated copper slot grids. After drying, these were stained in 1% aqueous uranyl acetate and Reynold's lead citrate, and then viewed in a JEOL JEM-1400 Plus TEM.

Representative images were collected on a GATAN OneView camera for analysis of GFB structural changes using Fiji, as similarly performed by Benchimol de Souza *et al.*^44^ The mean podocyte frequency per μm length (of basement membrane) and mean podocyte width were determined in all clearly identifiable sections of the GFB (Fig. 3A).

### Statistical analysis

All data were statistically analyzed and graphed with GraphPad Prism 7 (La Jolla, CA). All percentage data were arcsine transformed before analysis. Dextran clearance from the dorsal aorta, heart rate, and blood flow were subjected to two-way ANOVA. Foot process width and foot process number per μm GBM were analyzed by one-way ANOVA. Dextran excretion data were analyzed by linear regression and comparison of regression slopes. The first derivative of excretion data was determined for individual fish. Differences between the mean first derivative of control and treatment groups were determined for measures between 0–24 hpi and 24–48 hpi using *t*-tests. All means are reported with a standard error of the mean.

## Results

### Determination of doses for induction of pharmacological injury

The dose of a single intravenous injection of puromycin or adriamycin required to cause edema without effects on survival was tested. Achieving successful intravenous injections is considerably easier, and consequently more accurate, in 3 dpf fish due to the large CCV and SV. Due to a narrowing of the CCV by 4 dpf, injections in older larval fish are much more challenging and error prone. Adriamycin affects angiogenesis in mammals^45^ and, similarly in fish, affects formation of the subintestinal vessels and eye development.^46^ The measurement of FITC in peripheral vessels is therefore potentially problematic. Similarly, FITC measurements in the heart can be obscured by accumulation of dextran in the pericardial sac, particularly in cases of edema. To minimize any potential confounding effects on FITC measurements and to allow normalization to baseline values, measurements of FITC were determined in the dorsal aorta, as similarly performed by Rider *et al.*^40^

**FIG. 1.**
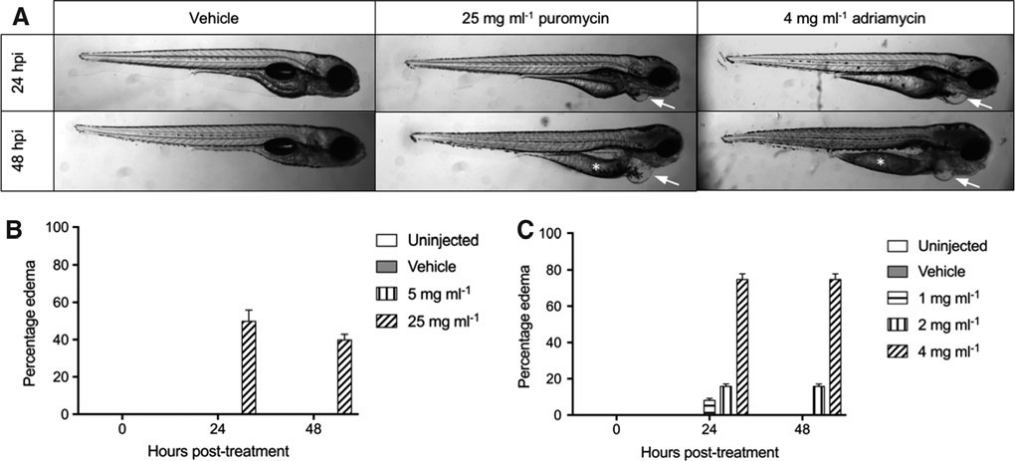
Determination of pharmacological injury doses—1 nL injection at 3 dpf*.* **(A)** Representative images showing pericardial edema (*arrows*) and remaining yolk reserves (*) presented in a portion of larvae administered adriamycin or puromycin. **(B)** Five milligrams per milliliter puromycin does not result in edema. Increasing the puromycin dose to 25 mg mL^−1^ causes edema in >40% of fish from 24 hpi (mean ± SEM). **(C)** Four milligrams per milliliter adriamycin causes edema in >75% of larvae by 24 hpi (mean ± SEM). SEM, standard error of the mean; dpf, days postfertilization; hpi, hours postinjection.

As podocytes exhibit elaborate primary processes and numerous interdigitating foot processes by 3 dpf,^26^ this age was selected for the injection of compounds to induce pharmacological injury. Edema is readily observed as a result of acute renal failure in larval fish.^21,39,40,47^ The concentration of puromycin was partially based on previous studies,^39^ and following preliminary studies, three concentrations of adriamycin were tested. One hundred percent survival was observed at both tested doses of puromycin (5 or 25 mg mL^−1^). Only the higher dose of puromycin resulted in edema (Fig. 1A), which was observed from 24 h in 45% of fish (Fig. 1B).

The higher doses of adriamycin (2 and 4 mg mL^−1^) decreased survival to 90% after 24 h. Two milligrams per milliliter adriamycin resulted in transient edema in >10% of fish, conversely 4 mg mL^−1^ resulted in persistent edema in 75% of larvae, some of which had remaining yolk reserves suggestive of stunted development (Fig. 1A, C). Due to the mortality and gross morphological changes associated with the highest dose of adriamycin, only 1 and 2 mg mL^−1^ concentrations were taken forward for further study.

### Effects of puromycin and adriamycin on renal permeability and cardiovascular function

Both puromycin^12^ and adriamycin^15^ are known to disrupt the GFB in mammals. While the effect of injected adriamycin on developed podocytes is untested in fish, puromycin has been shown to disrupt the fish GFB.^39^ Puromycin and adriamycin were compared as pharmacological podocyte injury models in larval fish by testing both target effects on glomerular permeability and side effects on cardiovascular function.

**FIG. 2.**
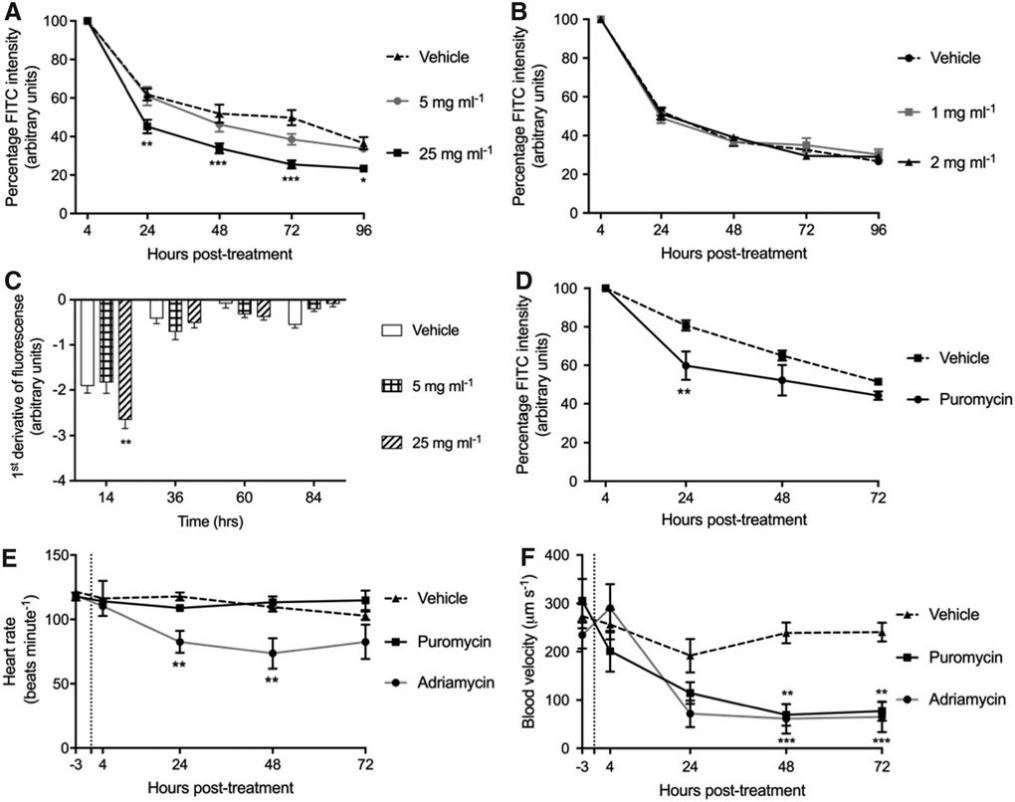
Comparison of adriamycin and puromycin as agents for podocyte injury. **(A)** Mean (±SEM) dextran clearance is significantly increased by injection of 25 mg mL^−1^, but not by 5 mg mL^−1^, puromycin at 3 dpf (*n* = *8*)*.* **(B)** Neither 1 nor 2 mg mL^−1^ adriamycin injected at 3 dpf has a significant effect on mean (±SEM) dextran clearance (*n* = *8*). **(C)** First derivative of **(A)** showing the rate change in dextran clearance is greatest within the first 24 hpi with the first derivative of 25 mg mL^−1^ puromycin being significantly greater than both controls and 5 mg mL^−1^-treated fish. **(D)** 25 mg mL^−1^ puromycin injected at 4 dpf also significantly decreases mean (±SEM) dextran clearance 24 hpi. **(E)** 2 mg mL^−1^ adriamycin significantly reduces mean (±SEM) heart rate, and puromycin has no effect (*n* = *8*)*.* **(F)** Mean (±SEM) venous blood flow is decreased by both 2 mg mL^−1^ adriamycin and 25 mg mL^−1^ puromycin (*n* = *8*). Two-way ANOVA, *p*-value summary: *<0.05; **≤0.002; ***≤0.0002.

Only the 25-mg mL^−1^ dose of puromycin resulted in increased clearance of injected 70 kDa dextran (Fig. 2A). Neither 1 nor 2 mg mL^−1^ adriamycin resulted in any increased clearance of 70 kDa dextran up to 96 hpi (Fig. 2B). The first derivative of puromycin-induced dextran clearance from the dorsal aorta shows that in all groups, the rate of change is highest over the initial 24 h postinjection (Fig. 2C). This is expected to be due to the continuing maturation of podocytes and the GFB structure between 3 and 4 dpf.^22,23,26,32^ Over the first 24 h, the 25-mg mL^−1^ dose of puromycin significantly increased the rate of loss of injected dextran compared with controls (Fig. 2C). The effect of increased dextran excretion by 25 mg mL^−1^ puromycin was also observed in fish injected at 4 dpf (Fig. 2D).

Despite having no effect on dextran clearance, the highest tested dose of adriamycin (2 mg mL^−1^) significantly decreased the heart rate (Fig. 2E) and blood flow (Fig. 2F). The highest dose of puromycin (25 mg mL^−1^) had no effect on heart rate, but reduced venous blood flow (Fig. 2F). Due to the lack of effect of adriamycin on dextran clearance and its side effects on heart rate and blood flow, this compound was not further assessed. Conversely, with its impact on dextran clearance, puromycin was further evaluated for its effects on podocyte ultrastructure.

### Analysis of puromycin-induced ultrastructural changes in the GFB

**FIG. 3.**
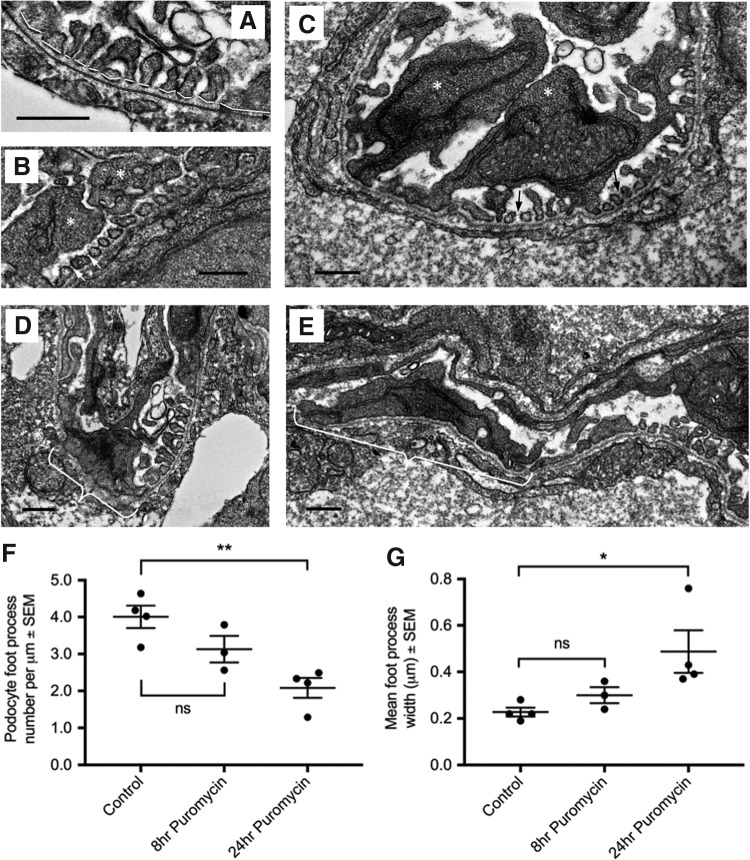
Effect of intravenous puromycin injection at 3 dpf on GFB structure (*n* = *3–4*)*.* **(A)** Image analysis of GFB for determination of podocyte effacement. *Black double line* = total length of GBM. *White lines* = foot process width. **(B, C)** Representative transmission electron microscopy micrographs at 24 hpi showing normal GFB structure with primary (*) and secondary foot processes with slit diaphragms (*arrows*). Foot process morphology is not significantly different from that of sham fish at 8 hpi (data not shown). **(D, E)** 25 mg mL^−1^ puromycin results in patchy podocyte effacement (*white brackets*). **(F)** Administration of 25 mg mL^−1^ puromycin significantly decreases the mean (±SEM) number of foot processes per unit length of GBM (*p* = 0.005) and **(G)** mean (±SEM) foot process width (*p* = 0.04). Analysis by one-way ANOVA with *post hoc* Dunnet's test. Scale bars are all 0.5 μm. GFB, glomerular filtration barrier; GBM, glomerular basement membrane. *p*-value summary: * < 0.05; ** ≤ 0.002.

To assess podocyte structure after 25 mg mL^−1^ puromycin injection, the ultrastructure of the GFB was quantitatively analyzed as performed by Benchimol *et al.* (Fig. 3A).^44^ Well-developed primary and secondary foot processes, SDs, and a trilaminar GBM were clearly visible at 4 dpf in healthy zebrafish glomeruli (Fig. 3B, C). Focal podocyte effacement, the loss of filtration slits, and enlargement of the foot process area in contact with the GFB were observed as a result of 25 mg mL^−1^ puromycin (Fig. 3D, E). To estimate the time point at which podocyte effacement occurs, samples were taken at 8 and 24 hpi. Foot process frequency (Fig. 3F) and foot process width (Fig. 3G) were not significantly altered until 24 hpi, suggesting that significant puromycin-induced podocyte effacement likely occurs between 8 and 24 hpi.

### Measurement of dextran excretion dynamics using a modified 96-well plate assay

**FIG. 4.**
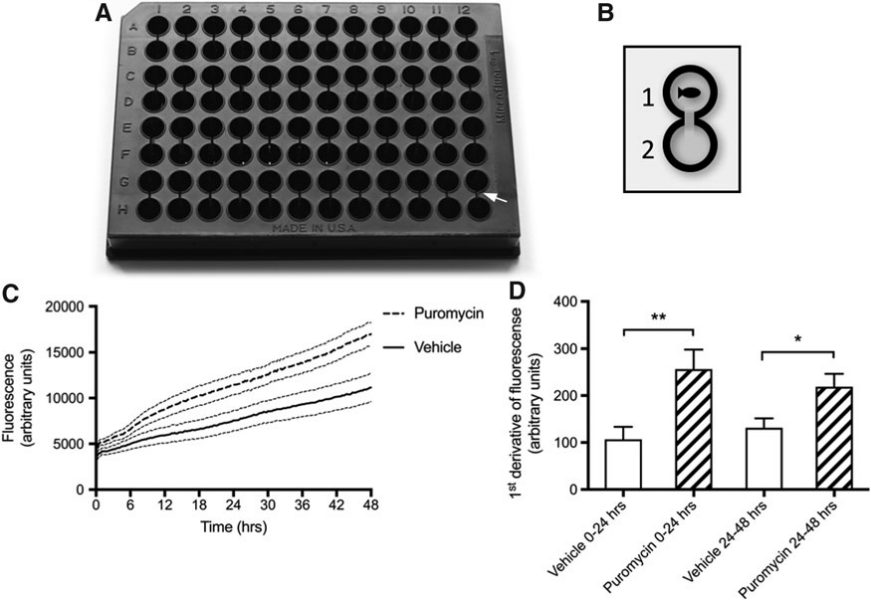
Plate assay to measure dynamics of cleared 70 kDa FITC-dextran following puromycin injection at 3 dpf (*n* = *14*)*.* **(A)** Photo of modified 96-well plate with 48 paired wells. *White arrow* pointing toward 250-μm slits between paired wells. **(B)** Schematic of well pairing. A single fish injected with FITC-dextran is placed in well 1. FITC fluorescence from excreted dextran is assayed in well 2. **(C)** Twenty-five milligrams per milliliter puromycin significantly increases mean (±SEM) clearance of 70 kDa FITC-dextran over 48 hpi. Analysis of linear regression slopes shows that both are significantly different (*p* = < 0.0001). **(D)** The mean (±SEM) first derivative of puromycin-treated fish between 0 and 24 hpi is 149 ± 49.4 higher than vehicle-treated fish. This is maintained, although to a lesser extent, over 24–48 hpi where the mean first derivative of puromycin-treated fish is 87.4 ± 34.2 higher than control fish. T-test, *p*-value: *<0.02; **<0.006.

Our validated puromycin injury model was used to test a novel assay we developed to minimize fish handling and to allow the dynamics of glomerular permeability to be measured in a semihigh-throughput manner. This method allows up to 48 fish to be studied simultaneously (Fig. 4A, B). The loss of glomerular selectivity by 25 mg mL^−1^ puromycin was also confirmed by our excretion assay (Fig. 4C). As similarly observed with the assay of FITC-dextran clearance from the dorsal aorta, the mean first derivative of the excretion assay shows that changes in dextran clearance are highest within 24 h of puromycin injection (Fig. 4D). This suggests that functional changes in the podocytes begin within 24 h postinjection.

## Discussion

The utility of acute pharmacological glomerular injury models would be greatly increased if they induced podocyte regeneration and the subsequent dynamics of GFB repair could be monitored. Unlike in human disease and experimental rodent models, zebrafish podocytes retain their regenerative capacity throughout their entire lifecycle.^35–37^ Current models of acute pharmacological podocyte injury in larval fish are limited by terminal edema. Edema is likely caused by a reduced renal flow, which impairs ion homeostasis due to a disruption in tubular ion uptake.^39,46,48^

To further the development of pharmacological glomerular injury methods in larval zebrafish, specific and off-target effects of puromycin and adriamycin were compared to identify a suitable compound for the induction and resolution of glomerular injury. To discriminate against effects arising from impaired podocyte development, podocyte toxins were administrated at 3 dpf. At this age, ∼70% the GBM is covered with organized podocyte foot processes and SDs, and the GFB is capable of selective filtraiton.^22,26,39^ To track the dynamics of glomerular function, we developed a novel assay to monitor the onset and resolution of abnormal protein filtration arising from podocyte injury and repair.

Rodent models of adriamycin nephropathy are well established and characterized by increased protein excretion, tubulointerstitial inflammation, and fibrosis.^15,49^ In larval fish, waterborne adriamycin administered from 9 to 48 h postfertilization (hpf) impairs the establishment of discriminative filtration due to abnormal podocyte development.^46^ Waterborne delivery of adriamycin is also associated with off-target toxicity, particularly to the heart.^15,46,50^ We aimed to reduce off-target effects by using a more direct delivery of adriamycin by intravenous microinjection, as similarly used in the rodent adriamycin nephropathy model.^15^ In our model assessing the effects of injected adriamycin in older larval fish with a functional glomerulus, the clearance of coinjected 70 kDa dextran was likely confounded by off-target cardiovascular effects.

The pronephric glomerular arterioles of zebrafish branch directly off the dorsal aorta, thus glomerular flow is tightly associated with flow in the central vasculature.^29,40^ In the present study, the adriamycin-induced reduction in heart rate and blood flow may have decreased glomerular flow rates and masked any increase in dextran clearance resulting from podocyte damage. Due to confounding off-target cardiovascular effects, adriamycin was considered unsuitable for modeling glomerular injury in zebrafish older than 3 dpf.

The injection of puromycin at 3 or 4 dpf in zebrafish larvae has been shown to result in podocyte effacement, loss of glomerular selectivity, and terminal edema.^39,51^ To mitigate off-target effects and edema, a lower dose of puromycin (5 mg mL^−1^) was tested, but was found insufficient for the measurable induction of glomerular injury. By injecting 1 nL of 25 mg mL^−1^ puromycin, as opposed to the 2.3–4.6-nL volume used by used Hentschel *et al.*,^39^ we observed similar effects on dextran clearance and podocyte effacement, including the onset of terminal edema. Injection volumes were restricted to 1 nL for the avoidance of negative effects on cardiovascular function, which can arise when injecting larger volumes into the circulation of larval fish (unpublished).

We employed image analysis^44^ to quantitate podocyte foot process width and foot process frequency. The quantification of podocyte morphology established that measurable structural changes occur between 8 and 24 hpi.

At a dose causing disruption of the GFB, puromycin also reduced blood flow by 24 hpi. Although decreased blood flow was observed, a higher dextran clearance was still detected, indicating a robust effect of puromycin on glomerular permeability. Previous studies have shown that FITC-dextran intensity in the vasculature is not significantly affected by edema-induced volume expansion.^39^ The dose of puromycin established for acute podocyte injury was not associated with any measurable effects on tubular structure (data not shown), including the brush border. The cilia of tubular cells that form the zebrafish nephron are essential for fluid clearance.^52^ This suggests that in the pronephros, effects of puromycin are specific to the glomerulus, as similarly reported by Hentschel *et al.*^39^

To advance methods for the *in vivo* analysis of glomerular permeability in zebrafish, we developed a novel assay of dextran excretion allowing the continuous monitoring of glomerular permeability to high-molecular-weight (HMW) molecules. Repeated handling and anesthesia of fish induce stress responses and cause detrimental effects on cardiovascular function.^41,53,54^ This limits the frequency and precision at which FITC-dextran clearance may be measured from the vasculature of larval fish. Hence, an advantage of our semihigh-throughput plate assay is that after injection of FITC-dextran, it involves no further handling or anesthesia of fish. This dextran excretion assay complemented measurements of FITC-dextran clearance from the dorsal aorta and confirmed that increased excretion of HMW molecules occurred in the first 24 h after injection of puromycin. While our dextran assays suggest that in some individuals glomerular function may resolve by 48 hpi, systemic effects leading to terminal edema were not resolved, as reported in previous studies.^39^

Puromycin- or adriamycin-induced nephrosis are among the oldest and most widely used models of human nephrotic syndrome, particularly using rats due to their susceptibility to both compounds.^55^ This study has identified the suitability of puromycin for induction of acute podocyte injury in zebrafish larvae. As observed in rodent models, the defined single dose of puromycin used in the present study results in podocyte effacement with a concurrent loss of glomerular selectivity. The onset of GFB changes within 24 h in fish is considerably faster than occurs in rodent models, which have a 4–5-day latency period after puromycin injection.^12,13,56^

In contrast to rodent models, puromycin administration in larval fish results in off-target effects on central blood flow, which may result in the terminal onset of edema.^39^ Further studies are required to assess the correlation between edema and GFB changes to establish if puromycin-induced injury is recoverable in larval fish, as observed in the genetic podocyte NTR-based podocyte injury model.^35,37^ A pharmacological glomerular injury model is yet to be established in adult zebrafish. Unlike larval fish, which can only repair their single paired nephron, glomerular injury in adult fish may induce neonephrogenesis, as documented with tubular injury.^36,57^

Our novel excretion assay for the monitoring of glomerular selectivity, a proxy of podocyte function, enables a semihigh-throughput and dynamic measurement of glomerular filtration without the confounding effects of repeated fish handling. This will facilitate the screening of small molecules that prevent glomerular injury or promote podocyte repair. The ability to measure the dynamics of GFB function provides an excellent tool for the *in vivo* analysis of glomerular injury and podocyte repair in larval zebrafish.
